# Who Cares about Biogenic Amines?

**DOI:** 10.3390/foods12213900

**Published:** 2023-10-24

**Authors:** Luigi Esposito, Dino Mastrocola, Maria Martuscelli

**Affiliations:** Department of Bioscience and Technology for Food, Agriculture and Environment, University of Teramo, Via R. Balzarini 1, 64100 Teramo, Italy; lesposito2@unite.it (L.E.); dmastrocola@unite.it (D.M.)

Biogenic amines (BAs) have been under study since the early 1970s [1]. Significant efforts have been made to understand the mechanisms of BAs formation in foods by microorganisms and other biochemical pathways [2,3,4]. This topic has garnered substantial knowledge, engaging experts from various fields, including biology, microbiology, food science and technology, biochemistry, and analytical chemistry. Nevertheless, it remains challenging to control BAs and to classify them as hazardous or not. New perspectives are also coming highlighting BAs role as for polyamines fundamental for human health than for food quality [5]. Generally, BAs are present in food and may be related to its spoilage (due to the incorrect storage and uncontrolled microbial activity). Moreover, they may cause adverse health concerns diminishing the human detoxifying system activity (intestinal aminoxidase enzymes) when individual sensitivity or other conditions are present or unusual levels of BAs are found in foods [6].

One of the primary challenges with BAs is their analytical determination. The available and tested methods typically demand highly skilled personnel and sophisticated equipment. The European Food Safety Authority (EFSA) recommends an extraction technique coupled with high-performance liquid chromatography (HPLC) as the official method for quantifying BAs [7]. However, this approach is time-consuming and does not allow for quick detection. Other proposed analytical methods include thin-layer chromatography, gas chromatography, and capillary electrophoresis [8,9].

In recent years, high-resolution nuclear magnetic resonance (HR-NMR) has been employed to monitor changes during storage and is generally used as a predictive tool in various products [10]. In addition, optical and colorimetric methods are gaining traction as effective indicators of BAs directly on packaging, serving as smart tools for food quality inspection [11]. All of these approaches aim to expedite laboratory analyses while maintaining the highest precision and accuracy in determination. Thus, all the emerging techniques suggested as useful for BAs analysis, should also be complemented by correlation analysis with sensory, physical, and nutritional quality.

BAs need to be thoroughly investigated, and despite the wealth of high-quality literature available, a cultural shift must occur, acknowledging that their presence in foods and beverages is inevitable. A stronger connection with health experts is encouraged to protect consumers or, at the very least, inform them of the potential effects of BAs. Even in this regard, we are at the forefront of exploration. Furthermore, innovative methods for evaluating BAs in food processing [4,12,13,14,15,16,17] or for novel ingredient employment should be highlighted [18]. Thus, future developments should involve a deeper study of the dietary effects on humans concerning BAs exposure and, ultimately, urging competent authorities to consider BAs levels in products other than fish and its derivatives, as outlined in Commission Regulation (EC) No. 2073/2005.

All contributions are welcomed to support decision-making bodies and to encourage involved companies to prioritize BAs control as a quality assurance objective.

## Data Availability

Not applicable.
